# Limited risk of Zika virus transmission by five *Aedes albopictus* populations from Spain

**DOI:** 10.1186/s13071-019-3359-1

**Published:** 2019-03-29

**Authors:** Mikel A. González, Márcio G. Pavan, Rosilainy S. Fernandes, Núria Busquets, Mariana R. David, Ricardo Lourenço-Oliveira, Ana L. García-Pérez, Rafael Maciel-de-Freitas

**Affiliations:** 1 NEIKER-Instituto Vasco de Investigación y Desarrollo Agrario, Derio, Bizkaia Spain; 2 Laboratório de Mosquitos Transmissores de Hematozoários, Instituto Oswaldo Cruz, Fundação Oswaldo Cruz (IOC/ FIOCRUZ), Rio de Janeiro, Brazil; 3 IRTA, Centre de Recerca en Sanitat Animal (CReSA, IRTA-UAB), Campus de la Universitat Autònoma de Barcelona, Bellaterra, Spain; 4 Instituto Nacional de Ciência e Tecnologia em Entomologia Molecular, Universidade Federal do Rio de Janeiro, Rio de Janeiro, Brazil

**Keywords:** *Aedes albopictus*, Zika virus, Spanish populations, Vector competence, Viral copies, RT-qPCR, Plaque assay

## Abstract

**Background:**

*Aedes albopictus*, the Asian tiger mosquito, is an exotic invasive species in Europe. It has substantial public health relevance due to its potential role in transmitting several human pathogens. Out of the European countries, Spain has one of the highest risk levels of autochthonous arbovirus transmission due to both the high density of *Ae. albopictus* and the extensive tourist influx from vector-endemic areas. This study aims to investigate the susceptibility of five *Ae. albopictus* populations from mainland Spain and the Balearic Islands to a Brazilian Zika virus (ZIKV) strain.

**Methods:**

The F1 generation of each *Ae. albopictus* population was orally challenged with a ZIKV-infected blood meal (1.8 × 10^6^ PFU/ml). At 7 and 14 days post-infection (dpi), mosquito bodies (thorax and abdomen) and heads were individually analysed through RT-qPCR to determine the infection rate (IR) and dissemination rate (DR), respectively. The saliva of infected mosquitoes was inoculated in Vero cells and the transmission rate was assessed by plaque assay or RT-qPCR on ~33 individuals per population.

**Results:**

The IR and DR ranged between 12–88%, and 0–60%, respectively, suggesting that ZIKV is capable of crossing the midgut barrier. Remarkably, no infectious viral particle was found in saliva samples, indicating a low ability of ZIKV to overcome the salivary gland barrier. A subsequent assay revealed that a second non-infective blood meal 48 h after ZIKV exposure did not influence *Ae. albopictus* vector competence.

**Conclusions:**

The oral experimental ZIKV infections performed here indicate that *Ae. albopictus* from Spain become infected and disseminate the virus through the body but has a limited ability to transmit the Brazilian ZIKV strain through biting. Therefore, the results suggest a limited risk of autochthonous ZIKV transmission in Spain by *Ae. albopictus*.

## Background

*Aedes albopictus* (Diptera: Culicidae), also known as the Asian tiger mosquito, is ranked as one of the world’s 100 most invasive species in the world [1]. This species has undergone a massive global expansion over recent decades facilitated by an effective network of air, ground and maritime human transportation, and the international trade of used tyres, lucky bamboo and flower pots, for instance [2]. So far, *Ae. albopictus* has been recorded in 27 European countries [3] since its introduction at the end of the 1970s in Albania [4]. A few years later, this species became established in Italy, and by late 1990s reached France [5], making its arrival to Spain imminent at that time. Hence, in 2004, one larva in a tree hole and an adult male of *Ae. albopictus* were collected in the backyard of a house located near Barcelona [6]. In the following years, the presence of *Ae. albopictus* along the Mediterranean coast and Balearic Islands was also reported, with an upsurge in Catalonia and Valencia Autonomous Communities [7, 8].

The establishment of *Ae. albopictus* in Europe raises public health concerns since it is a competent vector of at least 26 arboviruses, including dengue (DENV), yellow fever (YFV), chikungunya (CHIKV) and Zika (ZIKV) viruses [9]. Imported cases of CHIKV and ZIKV have been reported in European countries infested by *Ae. albopictus*, representing an alarming scenario that must not be overlooked [10, 11]. The role of *Ae. albopictus* as a vector of ZIKV has been confirmed by vector competence assays and by the detection of naturally infected specimens [12].

Since 2016, the European Union has reported about 2340 ZIKV-confirmed cases. Spain is the second most affected country in the continent with 320 imported cases, half of them reported in Catalonia [13], one of the most likely port-of-entries for ZIKV in Europe due to its high touristic flow. Considering the high density of *Ae. albopictus* in Spain, especially in the Mediterranean region, it is essential to ascertain the vector competence of its local populations in order to estimate the potential risk of autochthonous ZIKV transmission.

In this study, we assessed the ability of five Spanish populations of *Ae. albopictus* to become infected, disseminate and transmit a Brazilian ZIKV strain. We also evaluated whether a second non-infected blood meal in previously ZIKV-exposed females would affect the viral load in mosquito tissues.

## Methods

### Mosquito populations

**Fig. 1:**
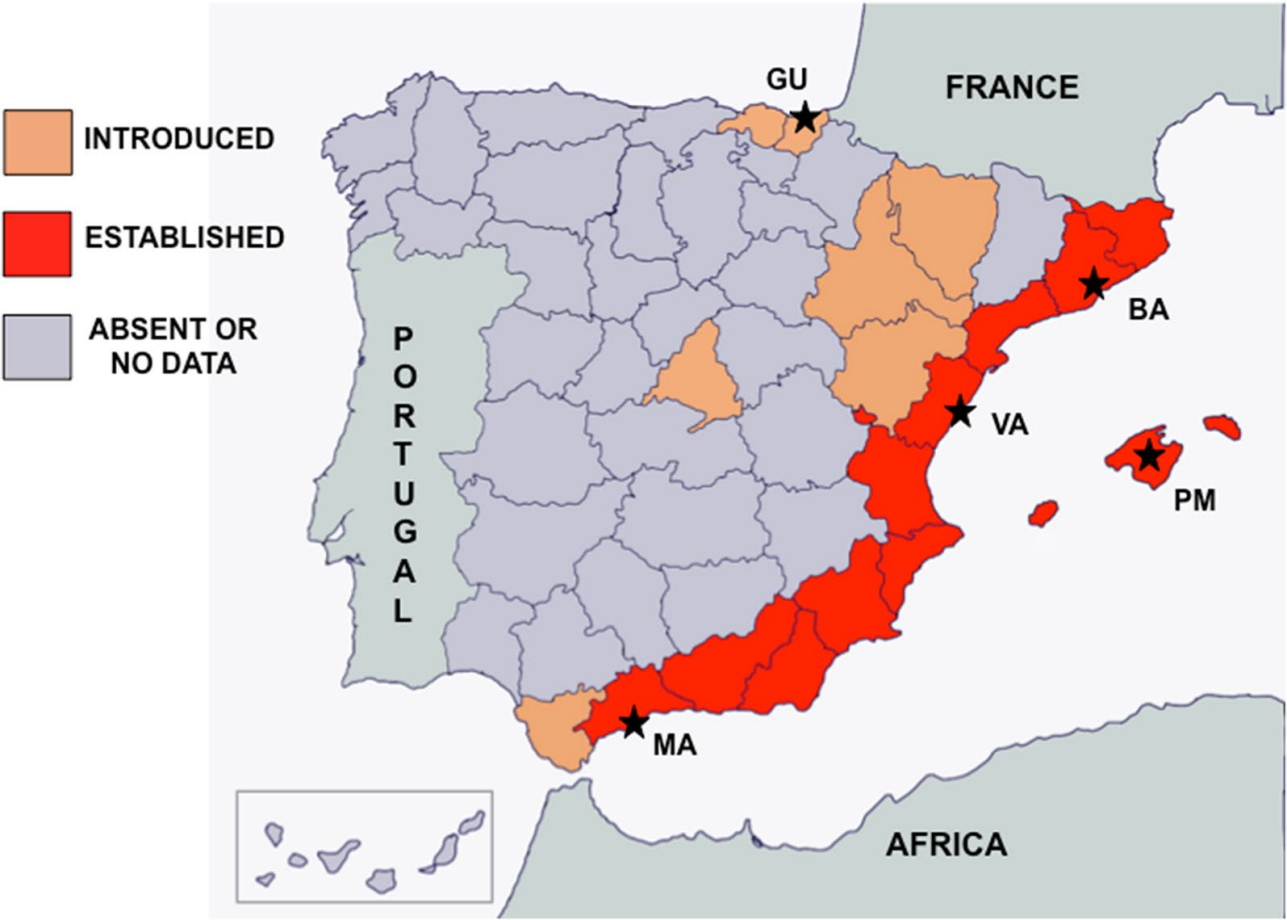
Geographical distribution of *Ae. albopictus* and sampled populations in the Iberian Peninsula and Islands. The five sampling sites are denoted with stars. The current distribution of *Ae. albopictus* is adapted from Collantes et al. [7], ECDC [14] and Alcibar et al. [15]. *Abbreviations*: BA, Barcelona; VA, Valencia; PM, Palma de Mallorca; MA, Málaga; GU, Guipúzkoa

Eggs of five *Ae. albopictus* populations from the Iberian Peninsula and Balearic Islands, from Barcelona (BA), Valencia (VA), Palma de Mallorca (PM), Málaga (MA) and Guipúzcoa (GU) were collected with ∼12 ovitraps/locality placed on urban or peri-urban sites during the summer of 2017 (Fig. 1, Table 1).

**Table 1:** Mosquito populations and field-collected eggs related information from Spanish mainland and Balearic Islands

Mosquito population	Collection site	Current status in Spain^a^	Total no. of eggs^b^	OPI^c^	EDI^d^
Barcelona, Catalonia (BA)	(41°48′33′′N, 2°03′33′′E)	Established for a long time	500	88.8	96.4
Valencia, Valencian community (VA)	(40°03′19′′N, 0°03′50′′E)	Established for a long time	1300	77.7	185.8
Palma de Mallorca, Balearic Islands (PM)	(39°41′45′′N, 2°42′0′′E)	Recently established	760	83.3	76.1
Málaga, Andalusia (MA)	(36°35′15′′N, 4°33′07′′W)	Recently established	450	87.5	64.4
Guipúzcoa, Basque Country (GU)	(43°20′35′′N, 1°45′44′′W)	Occasionally introduced	54	4.1	9.1

^a^Established for a long time (> 7 years) and recently established (< 3 years)

^b^Parental source of eggs (F_0_)

^c^Ovitrap Positive Index (OPI): frequency of positive ovitraps among the total examined

^d^Egg Density Index (EDI): no. of eggs / no. of positive ovitraps

Field-collected F_0_ generation was reared to F_1_ adult progeny to obtain enough mosquitoes to perform ZIKV oral infection experiments. *Aedes albopictus* females were fed twice per week on human blood (CAAE 53419815.9.0000.5248). Eggs were hatched, larvae were reared at a maximum density of ≈500 individuals/3 l of water in plastic trays and fed daily with fish food (Tetramin, Tetra, Melle, Germany) until pupation. Pupae were transferred to 30 × 30 × 30 cm cages (BugDorms, Taichung, Taiwan) to allow adult emergence and mating. Colony mosquitoes were maintained in an insectary at 27 ± 2 °C, 14:10 light:dark photoperiod, > 60% relative humidity and supplied with 15% sucrose diet *ad libitum*.

### Virus strain

The ZIKV strain (Asian lineage), namely ZIKV-PE243 (GenBank: KX197192), used in this study was isolated in 2015 from a patient from Recife, Pernambuco (northeast Brazil), [16] where a substantial increase of microcephaly cases in newborns was first detected in the Americas [17]. ZIKV was amplified in C6/36 mosquito cells (amplification step < 10) maintained with Leibovitz’s L15 medium (Sigma-Aldrich, Missouri, USA) supplemented with 2% fetal bovine serum (FBS, Gibco, Invitrogen, USA), and incubated for 7 days at 28 °C. Viral titers were quantified through a serial 10-fold dilution *via* plaque-forming assay in Vero cells (Sigma Aldrich, St. Louis, MO, USA) prior to experimental infection, as reported by Fernandes et al. [18]. The virus sample contained 1.8 × 10^6^ PFU/ml and was stored at -80 °C until use.

### Experiment 1: Vector competence of five *Ae. albopictus* populations from Spain

At 5–6 days post-emergence, about 500 females of each population were divided in groups of 65 specimens and transferred to cardboard feeding boxes without access to sucrose solution. Oral infection experiments were performed after 24 h with 6–7 day-old female mosquitoes using a membrane feeding system (Hemotek, Great Harwood, UK), adapted with a pig-gut covering. The infective blood meal was offered in a proportion of 1 ml of cell culture medium with virus to 2 ml of rabbit erythrocytes. Mosquito experimental infection was done at a Biosafety Level-2 laboratory. Oral feeding was limited to 30 min and only fully engorged females were selected for vector competence assays. Mosquitoes were held in cylinder plastic cups at 27 ± 0.5 °C, 12:12 h light:dark cycle with high relative humidity > 70%. Cotton balls with sucrose solution (15%) were provided on the top of the cups and replaced daily.

We analyzed 25 specimens of each of the five populations by RT-qPCR at 7 and 14 days post-infection (dpi) to determine the infection rate (IR) and dissemination rate (DR). The transmission rate (TR) was estimated by both plaque assay of saliva of 25 individuals at 7 and 14 days post-infection (dpi) per population and by RT-qPCR in 6–8 additional specimens per population at 14 dpi (Table 2).

**Table 2:** Summary of ZIKV vector competence experiments of *Ae. albopictus* populations from Spanish mainland and Balearic Islands

Mosquito population	No. of exposed females	Feeding rate (%)	Positive infected females (%)	Plaque assay^a^		Total no. of samples analysed	RT-PCR 14 dpi^b^
				7 dpi	14 dpi		
BA	500	52	88	25	25	150	8
VA	480	23	68	25	25	150	8
PM	392	26	24	25	25	150	6
MA	390	39	32	25	25	150	6
GU	442	39	84	25	25	150	8

^a^Plaque assay = 25 specimens per population (25 analysis of body, 25 analysis of head and 25 analysis of saliva) and condition

^b^Number of saliva samples analysed

*Abbreviations*: BA, Barcelona; VA, Valencia; PM, Palma de Mallorca; MA, Málaga; GU, Guipúzcoa

### Experiment 2: Effect of an additional uninfected blood meal on the *Ae. albopictus* vector competence to ZIKV

ZIKV-infected 6–7 day-old females were separated into two groups: one-time blood-fed (1BF) and twice blood-fed (2BF). After 48 h (a proxy to simulate a second blood ingestion on a natural setting), 2BF females were fed with an additional uninfected rabbit blood meal. Only 10 females from BA and 10 from GU fed twice from a total of 152 females (60 and 92, respectively). BA and GU were selected as representatives of different introduction histories in Spain, with the former established for a long time and the latter occasionally introduced. The viral RNA loads and rates were compared between 1BF and 2BF groups at 7 days after the first blood meal by RT-qPCR.

### Sample processing and virus detection by RT-qPCR

The head and body (thorax and abdomen) of the mosquitoes stored at -80 °C were dissected on a chill-plate, and 250 μl of Leibovitz’s L15 medium supplemented with 4% FBS was added to vials filled with 20–25 glass beads. Mosquito tissues were mechanically macerated using a rapid homogenizer (Precellys 24, Bertin Technologies, Montigny-le-Bretonneux, France) followed by centrifugation for 10 min at 10,000× *rpm* and at 4 °C. Suspended viral RNA was extracted from a 140 μl volume using QIAamp Viral RNA Mini Kit (Qiagen, Dusseldorf, Germany) following manufacturer’s instructions.

The one step RT-qPCR amplification (Superscript^TM^ III Platinum^TM^, Invitrogen, Waltham, MA, USA) was performed as previously described [19] with some minor modifications. In brief, PCR reactions were performed using 13.5 μl of master mix, 1.4 μl (10 μM) of each primer (forward: 5′-CTT GGA GTG CTT GTG ATT-3′; and reverse: 5′-CTC CTC CAG TGT TCA TTT-3′), 0.4 μl (50 mM) of MgSO_4_, 1.7 μl (10 μM) TaqMan TAMRA probe (FAM 5′-AGA AGA GAA TGA CCA CAA AGA TCA-3′ TAMRA), 0.4 μl of Taq polymerase, 5 μl of each RNA sample and 1.2 μl of ultra-pure water to a final volume of 25 μl. Amplification reactions were performed in a QuantStudio 6 Flex Real-Time PCR System (Applied Biosystems, Foster City, CA, USA) programmed as follows: 45 °C for 15 min and 95 °C for 2 min, followed by 40 cycles of 95 °C for 15 s, 58 °C for 5 s and 60 °C for 30 s.

### Collection of saliva and virus detection by plaque assay in Vero cells

Female mosquitoes at 7 and 14 dpi (Experiment 1) and at 7 dpi (Experiment 2) were immobilised on a Petri dish placed on ice and their legs and wings were removed with forceps. The proboscis of each live mosquito was inserted into a 10 μl pipet tip containing 5 µl of FBS and then expelled into a sterile 500 µl microcentrifuge tube with 45 µl of Leibovitz’s L15 medium supplemented with penicillin (10 μg/ml), gentamicin (1 μg/ml) and fungizone (1 μg/ml). After 30 min of salivation, saliva samples and mosquito bodies were immediately stored at -80 °C.

Vero cells were plated and incubated until monolayer formation. Then, the entire sample of the saliva (50 μl) for each mosquito was placed on a 6-well plate containing the cells and incubated for 1 h (37 °C, 5% CO_2_) and supplemented with Earle’s 199 medium. After 1 h incubation, the supernatant was removed, and the cells were overlaid with carboxymethyl cellulose (CMC) in Earle’s 199 medium. Inoculated Vero cells were incubated for 7 days and then fixed with 10% formaldehyde and stained with crystal violet (0.02%). To ensure cell viability and to prevent self-contamination during the process, 50 μl of 3-fold serial diluted ZIKV stock in Leibovitz’s L15 medium was added in every assay to one of the plates as a positive control and cell culture medium alone as a negative control.

### Viral quantification and statistical analysis

The number of ZIKV-RNA copies in the body and head was calculated with a ZIKV standard curve from 10-fold dilutions of known RNA copies (10^1^–10^9^) included on every PCR plate. The limit of virus detection by RT-qPCR was established in five RNA copies. IR and DR were calculated as the proportion of females with infected bodies among the total tested and the proportion of females with infected heads among the total tested, respectively. TR refers to the total number of females with viral particles in saliva among the total tested. To ease comparison with previous papers in which different mosquito populations were challenged for ZIKV [18–22], we sought to replicate the experimental design and estimate IR, DR and TR in infected mosquitoes. Using the same ZIKV isolate described here, vector competence (demonstrated by the presence of ZIKV particles in mosquito heads) ranged from 86–100%, highlighting good viral replication and the reliability of the techniques used for viral detection in varied tissues including mosquito saliva [21–23]. Fisher’s exact test was applied to detect significant differences between proportions (IR and DR). The number of viral copies was compared using the non-parametric Kruskall-Wallis test followed by pairwise comparisons. Mann-Whitney U-test was used to compare the viral titres between 7 and 14 dpi and between the groups of Experiment 2. Statistical analysis was performed with IBM SPSS statistics v.23.0.

## Results

### Indicators of *Ae. albopictus* infestation

A total 58 ovitraps were placed in the five regions from Spain (Table 1). Of these, 75% had at least one *Ae. albopictus* egg. The ovitrap positive index (OPI) ranged between 4–90% and the egg density index (EDI) ranged between 9–96 in July 2017. All geographical locations showed support for a high level of infestation by *Ae. albopictus* mosquitoes, except for the GU population, which showed a low frequency of positive ovitraps and a reduced number of eggs (Table 1).

### Viral competence parameters

**Fig. 2:**
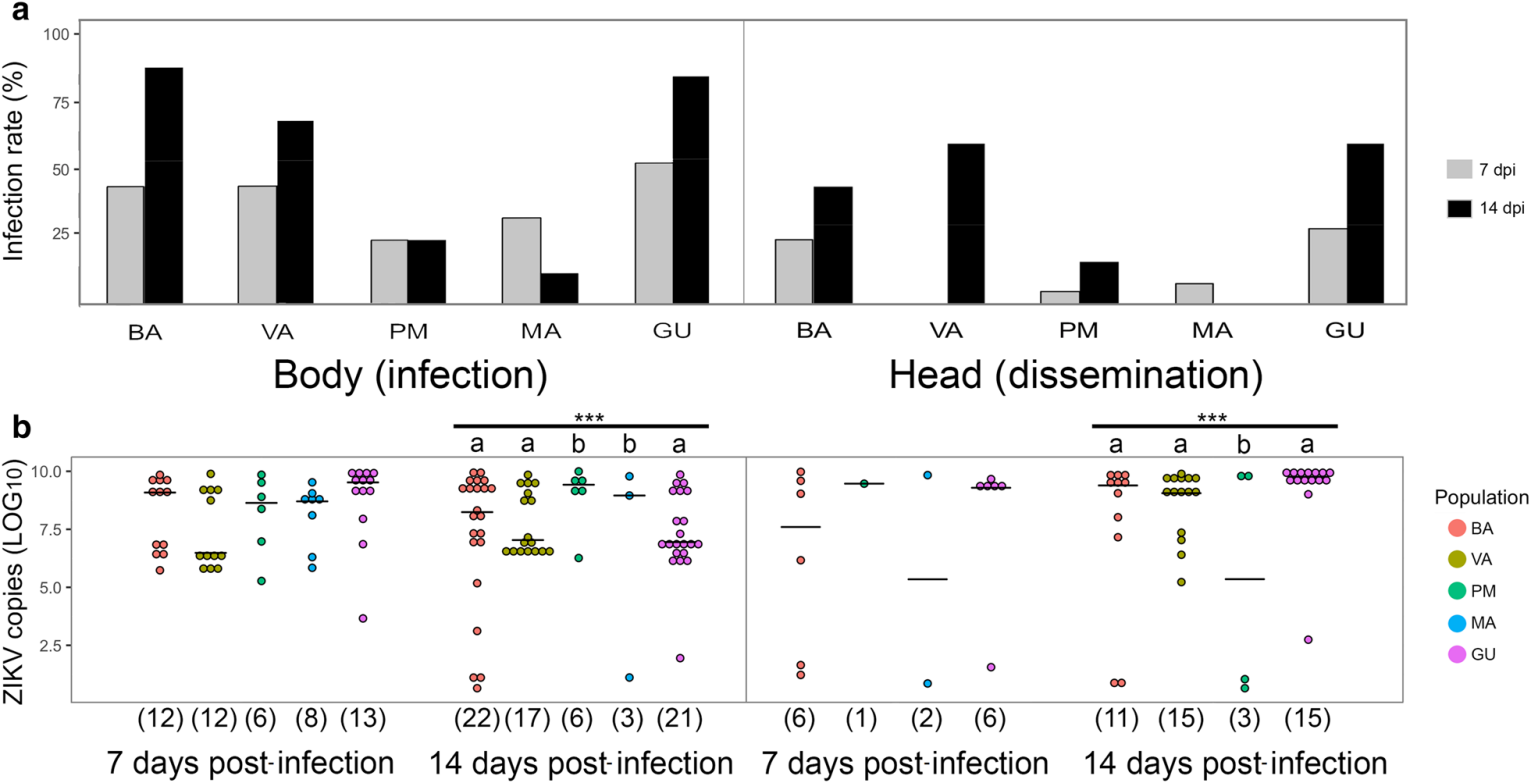
Viral competence parameters in the five Spanish *Ae. albopictus* populations. Infection and dissemination rates (**a**) and number of ZIKV RNA copies (**b**). Infection rates and absolute number of ZIKV copies in both body and head of the five *Ae. albopictus* populations from Spain at days 7 and 14 after oral experimental exposition with a ZIKV infectious blood meal (1.8 × 10^6^ PFU/ml). The five populations (BA, Barcelona; VA, Valencia; PM, Palma de Mallorca; MA, Málaga; GU, Guipúzcoa) are represented by different colours. Horizontal black bars represent the medians. The number of infected samples from the 25 mosquitoes analysed for each population are shown in parentheses. Different letters indicate statistically significant differences (Mann-Whitney U-test; ***P < 0.001)

In total, 38% of the females exposed to the infective blood meal were fully engorged (Table 2). Overall, the IR and DR increased at 14 dpi, except for the MA population for which a decrease was observed over time. Overall, the IR ranged from 39.2 to 55.2% and the DR varied from 13 to 36% at 7 and 14 dpi, respectively. A high level of heterogeneity was observed in the IR and DR among populations. BA, VA and GU populations showed superior permissiveness than PM and MA to ZIKV, with a higher DR at 7 dpi (Chi-square test, *χ*^2^ = 13.8, *df* = 4, *P* = 0.008) and IR and DR at 14 dpi (IR: *χ*^2^ = 48.8, *df* = 4, *P* ≤ 0.001 and DR: *χ*^2^ = 31.7, *df* = 4, *P* ≤ 0.001) (Fig. 2a). On the other hand, mosquitoes from PM and MA presented low values of IR and DR. No ZIKV-disseminated infection was observed in *Ae. albopictus* from VA at 7 dpi and from MA at 14 dpi (Fig. 2a). Remarkably, TR was zero for all populations as ZIKV particles were not detected in the saliva of any of the tested females by either plaque assay or RT-qPCR.

### Viral loads in mosquito tissues

At 7 dpi, viral copies in bodies were similar among populations, but more ZIKV copies were found in mosquito bodies from BA, VA and GU than in mosquitoes from PM and MA at 14 dpi (Kruskal-Wallis test, W = 49.9, *df* = 4, *P* ≤ 0.001) (Fig. 2b). On the other hand, the number of ZIKV copies in the heads at 7 dpi was not statistically different among populations. The number of viral copies in the body was heterogeneous at 14 dpi and specimens from PM were found with less ZIKV copies in the body compared to the other populations (Kruskal-Wallis test, W = 32.5, *df* = 4, *P* ≤ 0.0001) (Fig. 2b). Overall, the number of ZIKV copies had a 19-fold increase in infected heads (Mann-Whitney test, *U* = 1552.0, *P* = 0.207) and 13-fold in infected bodies (*U* = 216.0, *P* = 0.038) from 7 to 14 dpi.

### Effect of a second blood meal on vector competence

**Fig. 3:**
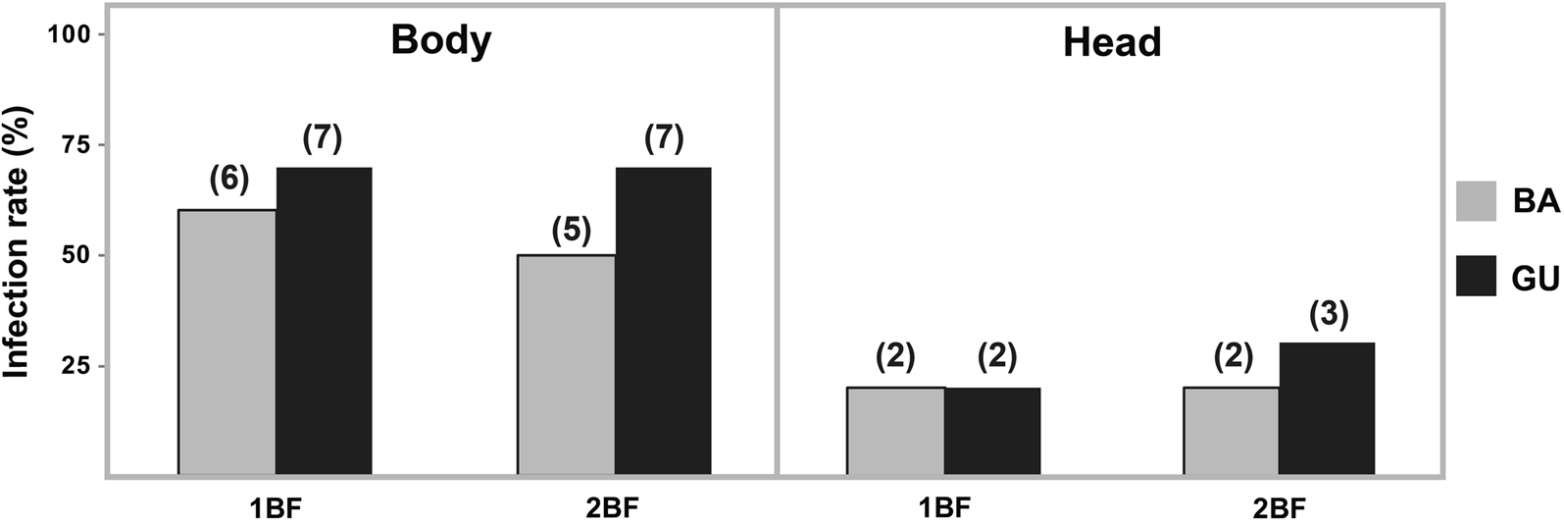
Comparison of ZIKV infection rates of *Ae. albopictus* populations exposed or non-exposed to an additional ZIKV non-infective blood meal. Infection rates (body and head) at 7 dpi of females that received a single ZIKV-infective blood meal (1BF) and those females fed on a second non-infective blood meal 48 h after viral exposure (2BF) are shown. Ten specimens were analysed for each condition. Populations: BA (Barcelona) and GU (Guipúzkoa). The numbers in parenthesis indicate the positive infected females

A second feeding on non-infected blood did not affect IR and DR (Mann-Whitney test, BA IR: *U* = 54.00, *P* = 0.781; BA DR: *U* = 55.00, *P* = 0.654; GU IR: *U* = 42.50, *P* = 0.293; GU DR: *U* = 26.00, *P* = 0.482) or number of viral copies (BA body: *U* = 46.00, *P* = 0.796; BA head: *U* = 45.00, *P* = 0.739; GU body: *U* = 44.50, *P* = 0.684; and GU head: *U* = 43.00, *P* = 0.631) (Fig. 3). Although not statistically significant, the mean ZIKV copies barely decreased in the BA population (1BF body: 2.3 × 10^5^ ± 1.3 × 10^5^; 2BF body: 3.0 × 10^4^ ± 2.5 × 10^4^; 1BF head: 78.4 ± 76.7; 2BF head: 5.1 ± 3.9 for 2BF), while the GU population exhibited an opposite outcome (1BF body: 8.7 × 10^5^ ± 7.0 × 10^5^, 2BF body: 3.7 × 10^6^ ± 2.8 × 10^6^; 1BF head: 34.4 ± 29.6; 2BF head: 1.4 × 10^5^ ± 1.1 × 10^5^). Thus, an additional blood-feeding did not influence vector competence of Spanish *Ae. albopictus* to ZIKV.

## Discussion

Vector competence is a key feature to determine the risk of pathogen transmission by mosquitoes. The success of a given mosquito population in transmitting a virus will depend on the ability of the virus to overcome both the midgut and salivary gland vector barriers [24]. Herein, we report the susceptibility of recently introduced and long-established *Ae. albopictus* populations from Spain to ZIKV. These five populations represent the current *Ae. albopictus* distribution in the country, portraying a nationwide sampling effort. Taken together, the results presented here suggested that this ZIKV strain is not transmitted during female blood-feeding due to the absence of infectious viral particles in *Ae. albopictus* saliva at 7 and 14 dpi.

*Aedes albopictus* has colonized the majority of the Spanish Mediterranean area since its arrival in 2004 [7] and its geographical distribution is still expanding with recent introductions in the Basque Country and Aragón [14]. According to the frequency of positive ovitraps and egg density retrieved from our data, the infestation index can be considered as high as in the regions where *Ae. albopictus* has been established for a long time or was recently introduced, which must concern public health managers. The exception is the Basque Country, where *Ae. albopictus* eggs were occasionally detected in restricted places near the border with France. In that particular case, public health authorities from the Basque Country must enhance vector surveillance and control on the border to avoid *Ae. albopictus* establishment.

The vector competence of *Ae. albopictus* to ZIKV has hitherto shown heterogeneous results, with the susceptibility status of field populations oscillating dramatically [12, 25, 26]. Our study has shown that Spanish *Ae. albopictus* populations are heterogeneously capable of becoming infected and disseminate ZIKV, but none were competent to transmit this virus strain through biting. The same ZIKV strain used herein showed high replication rates and was detected in *Ae. aegypti* saliva, demonstrating a high transmission rate [21, 22]. On the European scale, *Ae. albopictus* from France, Italy and Germany have demonstrated variable susceptibility to Asiatic ZIKV lineages [10, 27–29]. Our findings are aligned with those reported for *Ae. albopictus* populations from France and Italy [10, 29], which showed only few specimens with ZIKV in the saliva. Altogether, these results reinforce the hypothesis that *Ae. albopictus* from Mediterranean Europe might have limited vectorial capacity, at least for the ZIKV strain circulating in Brazil. Nonetheless, conclusions regarding the absence of ZIKV in *Ae. albopictus* saliva must be taken carefully for at least two reasons. First, forced salivation is a common method to diagnose arbovirus presence in saliva, but its collection is obtained after mosquitoes are immobilized with legs and wings removed. Therefore, the use of non-sacrificial methods to collect saliva such as using a strip of filter paper, must be encouraged for vector competence studies [30]. Secondly, and most importantly, considering a mosquito species a negligible vector might produce undesired consequences such as the relaxation of vector control.

The viral concentration used in our study was slightly lower compared to other studies conducted with European populations of *Ae. albopictus* [10, 29], but with more viral particles than are naturally found in infected hosts [12]. In addition, environmental factors such as temperature may affect vector competence. For instance, the susceptibility of *Ae. albopictus* for CHIKV is affected depending on the incubation temperature of immature stages [31, 32]. Moreover, our infection was conducted with fresh-wild populations (F1 generation), which might reflect better the vector competence of field populations [33].

Besides suitable environmental conditions, the establishment of an arbovirus transmission cycle depends on the genetic background of mosquito populations and the viral genotype [34, 35]. In Spain, two main hypotheses of *Ae. albopictus* introduction have been proposed, but not confirmed. The first is the dispersion along the entire Spanish Mediterranean coast including the Balearic Islands since their introduction and establishment in Barcelona. The second possible way of arrival is by occasional introductions from France to Guipúzkoa (northeast Spain) [7, 8]. In this scenario, mosquitoes from the Guipúzkoa population may proceed from a non-established population, while the Barcelona and Valencia females might come from a long-established one. Despite that, GU, BA and VA exhibited higher IR and DR,  whereas Palma de Mallorca and Málaga populations (collected in areas recently colonized by *Ae. albopictus*) showed an overall lower virus infection and dissemination. This lower vector competence could be explained by an impairment between mosquito and virus genotypes. The validation of this hypothesis depends on increasing the number of tested samples, future research on population genetics of these mosquitoes and assessing the vector competence of local *Ae. albopictus* for different strains of ZIKV but also other arboviruses. Until then, vector surveillance authorities must remain alert to produce a rapid response and effective vector control when suitable.

*Aedes albopictus* may feed on blood more than once in a single gonotrophic cycle [36]. Hence, understanding if repeated blood-feeding improves the viral load and infection rates is relevant from an epidemiological point of view, as the risk of arbovirus transmission might increase with repeated contact with hosts. Although the results of Experiment 2 were disparate between populations, it did not show statistically significant differences in viral copies after infected mosquitoes received a second non-infective blood meal. *Aedes albopictus* females from the PV population had a slightly increased viral load after the second blood meal, while the opposite was observed for BA. Amuzu et al. [37] evaluated the effect of multiple blood meals on dengue dynamics in *Ae. aegypti* and concluded that repeat feeding did not lead to an increase of IR or viral loads. Interestingly, a second non-infective blood meal shortened the extrinsic incubation period (EIP) of ZIKV-infected *Ae. albopictus* [38]. Our data did not support the increased percentage of mosquitoes observed by Armstrong et al. [38] who offered the second blood meal 4 days after ZIKV infection; in our study the second blood meal was offered only 48 h after infection. Blood-feeding triggers mechanical and physiological changes in mosquitoes that could affect virus replication and thus the EIP. Therefore, it is possible that the effects of a second blood meal on virus replication is dependent on the time after infection it is offered to mosquitoes [39].

Furthermore, the vector potential of other species to transmit ZIKV in Spain should also be considered. The recently sporadic incursion of *Ae. aegypti* supported by maritime transport represents a recent rising concern in Spain. In December 2017, *Ae. aegypti* mosquitoes were reported in the Spanish outermost region of the Canary Islands, a popular destination for travellers [40]. Indeed, the new arrival and or/establishment of other exotic mosquito species of *Aedes*, i.e. *Ae. japonicus*, in a rural area of north Spain, suggests that the Spanish epidemic potential to arboviruses is dynamic and could change underlying the rapid global spread of invasive species [41].

## Conclusions

The results from the oral experimental infections carried out in the present study suggest that Spanish *Ae. albopictus* populations are capable of becoming infected and disseminating ZIKV to other tissues. However, *Ae. albopictus* seems to have a limited ability for transmitting the ZIKV strain used herein since no viral particles were detected in saliva. Additional vector competence assays with other ZIKV strains must be conducted to increase the confidence of appointing Spanish *Ae. albopictus* as a non-competent vector.

## Abbreviations

CHIKV: chikungunya virus
CMC: carboxymethyl cellulose
C_T_: cycle threshold
FBS: fetal bovine serum
DENV: dengue virus
dpi: days post-infection
DR: dissemination rate
EDI: egg density index
EIP: extrinsic incubation period
IR: infection rate
OPI: ovitrap positive index
PFU: plaque-forming unit
PCR: polymerase chain reaction
RT-qPCR: reverse transcription qPCR
TR: transmission rate
YFV: yellow fever virus
ZIKV: Zika virus
